# The Influence of Perceived Physician Guidance and Trust on Early‐Career Nurse Practitioners’ Clinical Leadership: A Cross‐Sectional Mixed‐Methods Study

**DOI:** 10.1155/jonm/1900444

**Published:** 2026-11-03

**Authors:** Guoyang Zhang, Hester W. H. Smeets, Carolin Sehlbach, Frank W. J. M. Smeenk, Renée E. Stalmeijer

**Affiliations:** ^1^ Department of Educational Development and Research School of Health Professions Education Faculty of Health Medicine and Life Sciences Maastricht University Maastricht the Netherlands maastrichtuniversity.nl; ^2^ Research Centre for Interprofessional Collaboration in the Community Zuyd University of Applied Sciences Heerlen the Netherlands zuyd.nl; ^3^ Catharina Hospital Eindhoven the Netherlands catharinaziekenhuis.nl

**Keywords:** clinical leadership, interprofessional collaboration, nurse practitioner, physician guidance, physician trust, workplace learning

## Abstract

**Background:**

Increasing healthcare pressures have accelerated the integration of advanced practice providers, such as nurse practitioners (NPs), into healthcare teams. NPs work at the interface of medical and nursing domains by coordinating care and facilitating interprofessional collaboration. To perform this role effectively, NPs must develop clinical leadership. However, limited research has examined clinical leadership among NPs and how physician guidance and trust are perceived to influence it.

**Aims:**

To examine clinical leadership among early‐career NPs, assess its associations with perceived physician guidance and trust, and explore how NPs perceive these factors as shaping their clinical leadership.

**Design:**

This was a cross‐sectional, embedded, correlational mixed‐methods study.

**Methods:**

A total of 222 early‐career NPs in the Netherlands participated in the study. Quantitative analyses included descriptive statistics, group comparisons, multiple linear regression, and mediation analyses, while open‐ended responses were analyzed using a semantic approach to thematic analysis.

**Results:**

NPs reported moderate to high levels of clinical leadership. First‐year student NPs reported lower clinical leadership scores, whereas NPs with four to five years of practice reported higher scores. In multivariate analyses, perceived physician guidance was positively associated with clinical leadership, whereas perceived physician trust was not directly associated with clinical leadership. Mediation analyses indicated an indirect effect between perceived physician trust and clinical leadership via perceived physician guidance. The qualitative findings complemented these results: NPs described physicians’ emotional and practical support, constructive feedback, and leadership opportunities as enhancing NPs’ confidence and professional legitimacy. They also perceived physician trust as fostering psychological safety through which perceived physician guidance could support their clinical leadership.

**Conclusion:**

These findings underscore the importance of physician guidance and trust‐based relationships as relational conditions that may support clinical leadership among early‐career NPs.

**Summary**
• Implications to Nursing Management
◦ This study provides practical implications for supporting clinical leadership among early‐career NPs. NP education programs could incorporate explicit clinical leadership content into their curricula to prepare NPs for progressively greater responsibility. This preparation could be reinforced in clinical workplaces through structured supervision during training and early practice, with opportunities for NPs to observe physicians’ leadership practices, receive constructive feedback, and practice leadership‐related tasks in a supportive environment. To strengthen these workplace learning conditions, preparation of physicians for supervisory roles could be incorporated into continuing professional development, with attention to developmentally appropriate guidance, constructive feedback, and the provision of appropriate leadership opportunities. In addition, trust‐based relationships also appeared to be an important condition for clinical leadership. Physicians may cultivate such relationships by involving NPs in clinical reasoning and recognizing their contributions within teams. NPs, in turn, may build physicians’ trust by demonstrating clinical preparedness, communicating openly about uncertainties, and proactively seeking feedback.

## 1. Background

Globally, healthcare is facing significant challenges including physician shortages, the rising prevalence of chronic diseases, and strained healthcare resources [1, 2]. These pressures have accelerated a rise in the utilization and integration of advanced practice providers like nurse practitioners (NPs) into healthcare teams [1, 3]. NPs are registered nurses with advanced education and clinical training who can diagnose illnesses, order and interpret diagnostic tests, as well as prescribe medications within their scope of practice [4–8]. By bridging the medical and nursing domains, NPs are uniquely positioned to coordinate patient care with different health professionals to enhance interprofessional collaboration (IPC) [7, 9–14]. In order to execute this bridging role, NPs need to assume *clinical leadership*, defined as the process where they influence team members to provide high‐quality, patient‐centered care [13–15]. In practice, NPs’ clinical leadership can take the form of coordinating care, advocating for patient goals during team decision‐making processes, supporting colleagues, and initiating improvements in care [13–15].

However, developing clinical leadership is not straightforward. For NPs, clinical leadership development mainly occurs in the workplace through learning by doing, and such learning is typically informal and implicit [13–15]. From a sociocultural learning perspective, NPs’ clinical leadership development can be understood as a socially constructed process that takes place through participation in NPs’ communities of practice and through engagement across the broader landscape of practice [16, 17]. In IPC processes, which constitute a central aspect of the workplace learning environment for NPs, NPs interact with many different healthcare professionals [7, 9, 10, 12–15, 18]. Among these professionals, physicians are key figures who shape NPs’ learning experiences and professional trajectories [19–23]. Previous research has already indicated that physicians provide guidance to NPs, such as support with diagnostic and treatment decisions as well as feedback on clinical practice [20–23]. Drawing on sociocultural learning theories [16, 17], such guidance can be understood as relational, work‐embedded practice through which more experienced professionals support others’ learning and participation [24–26]. Through this guidance, physicians may help NPs develop the competence and confidence required to enact clinical leadership [20–23]. Yet this developmental process is also shaped, and at times constrained, by traditional healthcare hierarchies [27–32]. As the traditional leaders of clinical teams, physicians’ influence on the IPC may affect the extent to which NPs are afforded the trust and empowerment necessary for assuming leadership responsibilities [27–33]. This dynamic creates a critical tension: while physicians’ guidance may support NPs’ workplace learning and clinical leadership, hierarchical structures may simultaneously limit NPs’ opportunities to practice and develop clinical leadership [28, 34, 35].

To date, existing research offers limited insight into clinical leadership among early‐career NPs and its relationship with perceived physician guidance and trust. This is particularly relevant during training and the early stages of NP careers when NPs are assuming their professional roles and learning to exercise clinical leadership [12]. Understanding these relationships is important for identifying workplace conditions that may support clinical leadership among early‐career NPs. This study therefore examines clinical leadership among early‐career NPs, including those in training and those with up to five years of practice experience, investigates its associations with perceived physician guidance and trust, and explores how NPs perceive both factors as supporting their clinical leadership. The overarching research question was: How are perceived physician guidance and trust related to clinical leadership among early‐career NPs, and how do NPs describe these relationships? To address this overarching question, three sub‐research questions shaped the study: (1) What is the extent of clinical leadership as reported by early‐career NPs? (2) How is early‐career NPs’ clinical leadership associated with perceived physician guidance and trust? (3) How do early‐career NPs describe guidance and trust from physicians as supporting or constraining their clinical leadership?

## 2. Methods

We employed an embedded, correlational mixed‐methods design, in which quantitative data were primary and qualitative data provided contextual insights [36, 37]. Quantitative and qualitative data were collected concurrently from early‐career NPs using a survey comprising both closed‐ and open‐ended questions. The closed‐ended questions assessed early‐career NPs’ clinical leadership and the influence of perceived physician guidance and trust on NPs’ clinical leadership. Complementarily, the open‐ended questions explored how NPs enacted clinical leadership in practice and how they perceived physician guidance and trust as supporting or constraining their clinical leadership.

### 2.1. Setting

This study was conducted in the Netherlands, where registered nurses with a four‐year nursing bachelor’s degree and at least two years of working experience can enroll in a two‐year master’s program in advanced nursing practice to become an NP [38–40]. The master’s program combines coursework with training at the workplace, offering two specializations: general health care and mental health care. Student NPs and NPs work across diverse settings, including primary care, hospital wards, outpatient clinics, community health services, and mental‐health institutions. Within the training program, apprenticeships typically involve structured supervision by physicians and/or senior NPs, with the intention of fostering clinical reasoning, professional autonomy, and clinical leadership [41].

### 2.2. Participants and Procedures

Student NPs and NPs with up to five years of clinical experience were considered eligible. The research team contacted institutional contact persons, who facilitated recruitment of participants. To maximize the response rate, up to five reminder emails were sent from July to November 2025. At one institution, paper questionnaires were also distributed to facilitate participation among student NPs to increase initial low response. All responses were anonymized.

### 2.3. Questionnaire Development and Validation

Because no existing instrument was identified that directly assessed NPs’ clinical leadership, an adapted questionnaire was developed for this study. The adaptation was informed by the Leadership Practices Inventory (LPI), developed by Kouzes and Posner (2017) [42], and by previous applications of the LPI in nursing leadership research [43–49]. The LPI identifies five dimensions of leadership practice: *Model the Way, Inspire a Shared Vision, Challenge the Process, Enable Others to Act, and Encourage the Heart* [42]. The LPI was used as an organizing framework for the adapted questionnaire. Although NPs share some clinical leadership behaviors with nurses, their clinical leadership also involves advanced assessment, independent decision‐making, and responsibility for diagnosis and treatment. The original LPI items were therefore reviewed and adapted, with reference to the literature on NP clinical leadership [13–15], to reflect observable behaviors through which clinical leadership might be enacted in NP practice. For example, the LPI item “*Actively searches for innovative ways to improve what we do*” was adapted to “*I use evidence-based reasoning to improve patient care*” to reflect the emphasis on evidence‐based clinical reasoning within NP practice. Thereafter, the items of the initial adapted questionnaire were translated to Dutch using DeepL and pilot tested with three student NPs and two practicing NPs. Using think‐aloud and verbal probing techniques [50], HWHS and FWJMS explored pilot participants’ interpretations, clarity, and comprehension of the questionnaire items. Feedback from these sessions was used to refine item wording for precision and contextual relevance. Responses were recorded on a five‐point Likert scale (1 = Almost never; 5 = Almost always). Exploratory factor analysis was used to examine the underlying structure of the clinical leadership questionnaire, given that its factor structure had not been established in this population. Analyses were conducted in R (version 4.5.1) [51] using the lavaan package [52]. An oblimin rotation was applied to allow for correlations among factors, which was considered appropriate given the theoretically related dimensions of clinical leadership. Factor correlations (Φ) ranged from 0.34 to 0.43, indicating moderate associations among the latent dimensions. Of the 20 initial items, 8 did not meet the factor‐retention criteria and were excluded, resulting in a 12‐item, three‐factor solution (Supporting Table S1–3). The three factors were labeled “*visionary and inspirational role modeling*,” “*empowering and supporting others*,” and “*reflective and development-oriented practice*.” Internal consistency estimates were acceptable (Cronbach’s *α* = 0.58–0.79), and factorial stability was moderate to high (Tucker’s *φ* = 0.66–0.92), providing partial support for the reproducibility of the factor structure (Supporting Table S4–5). This three‐factor structure differed from the original five‐factor LPI, possibly reflecting the substantial contextual adaptation and item reduction undertaken in this study, as well as differences in how clinical leadership behaviors were expressed among early‐career NPs.

For guidance, items were informed by research on workplace guidance and by cognitive apprenticeship and sociocultural learning theories [16, 17]. Items capturing feedback and support were included, as these dimensions reflect central mechanisms through which physicians may influence NPs’ clinical leadership [24]. Confirmatory factor analysis (CFA) of the six‐item perceived physician guidance scale was conducted using the lavaan package [52] in R (version 4.5.1) [51]. The two‐factor model showed a good fit to the data (*χ* ^2^(8) = 31.99, *p* < 0.001; CFI = 0.99; TLI = 0.98; RMSEA = 0.05; SRMR = 0.03). Standardized factor loadings ranged from 0.82 to 0.86 for support and from 0.92 to 0.94 for feedback, all statistically significant (*p* < 0.001). The two latent factors were strongly correlated (*r* = 0.68, *p* < 0.001), suggesting that they represent related aspects of perceived physician guidance.

The complete set of questionnaire items is provided in the Supporting Information.

### 2.4. Quantitative Analysis

Descriptive statistics were computed to summarize participant characteristics and key study variables. Bivariate analyses, including *t*‐tests and one‐way ANOVAs, were performed to explore differences in clinical leadership scores across demographic and professional subgroups (e.g., age, stage of career, previous nursing experience, specialization, work setting). Spearman’s correlation coefficients were calculated to examine associations among perceived physician guidance, perceived physician trust, and clinical leadership. Multiple linear regression analysis was conducted to examine factors associated with clinical leadership. Perceived physician guidance and trust were entered as the main independent variables of interest, while age, career stage, previous nursing experience, specialization, work setting, team size, team affiliation, and perceived role clarity were included as covariates. Model assumptions were checked for linearity, multicollinearity, and normality of residuals. Finally, a mediation analysis [53] using bootstrapping procedures (1000 resamples) was performed to test whether perceived physician guidance mediated the relationship between perceived physician trust and NPs’ clinical leadership. The average causal mediation effect (ACME), average direct effect (ADE), and total effect were estimated with 95% confidence intervals, and statistical significance was set at *p* < 0.05.

### 2.5. Qualitative Analysis

The survey contained four open‐ended questions asking participants to provide examples of how they enacted clinical leadership in everyday practice and to describe how perceived trust, guidance, and insufficient guidance from physicians affected their enactment of clinical leadership. Response rates for the four questions were 92.3% (205/222), 88.7% (197/222), 86.5% (192/222), and 82.0% (182/222), respectively. The corresponding responses in the original Dutch dataset contained 4,433, 3,284, 3,083, and 2743 words, resulting in a total dataset of 13,543 words.

The open‐ended responses were analyzed using a semantic approach to thematic analysis informed by Braun and Clarke’s six‐phase process. A semantic approach was selected because the relatively brief responses provided limited contextual depth; the analysis therefore focused on participants’ explicit, surface‐level meanings [54]. The Dutch responses were translated into English using DeepL and reviewed by the research team for accuracy before analysis. The analysis combined deductive and inductive approaches. The research questions provided a deductive analytical framework focusing on the enactment of clinical leadership and the perceived roles of physicians’ guidance and trust. Within this framework, initial codes and categories were developed inductively from participants’ responses and subsequently organized into two overarching themes: (1) enacting clinical leadership in practice and (2) perceived physician guidance and trust as supporting or constraining clinical leadership. First, GYZ read and reread the complete dataset to become familiar with the responses. GYZ and CS then independently coded the same subset of responses. The initial codes and categories were compared and discussed with the wider research team to develop a preliminary codebook. For example, the statement “*trust gives me the confidence to raise issues for improvement*” was coded as “*confidence to speak up*,” grouped under the category “*psychological safety*,” while the codes and categories were iteratively reviewed and refined through team discussions. Finally, the qualitative findings were integrated with the quantitative results.

### 2.6. Reflexivity

The research team consisted of one PhD candidate in workplace learning and IPC (GYZ); a qualitative researcher at a university of applied sciences specializing in IPC, who had regular contact throughout the study with an NP colleague who coordinated an NP educational program (HWHS); a health scientist with expertise in workplace learning and patient feedback (CS); a pulmonologist with extensive experience in collaborating with and training NPs (FWJMS); and an educational scientist specializing in workplace learning, with experience in training NP workplace supervisors, including physicians and NPs (RS). The multidisciplinary composition of the team enhanced the reflexive rigor of the study. FWJMS contributed clinical insights into physician and NP collaboration, while CS, RS, and HWHS provided theoretical and methodological perspectives. HS, FWJMS, and RS enabled data collection through their networks and familiarity with the Dutch NP training institutes.

### 2.7. Ethical Approval

The institutional ethical review board of the Faculty of Health, Medicine and Life Sciences of Maastricht University approved the research (reference number FHML‐REC/2024/078). Informed consent was requested in the questionnaire. Participation in the study was anonymous and voluntary.

## 3. Results

The findings are presented in three parts. First, descriptive statistics summarize the participant characteristics. Second, descriptive analyses present the extent of clinical leadership reported by early‐career NPs, while group comparisons examine differences in clinical leadership across participant characteristics. Relevant qualitative findings are used to elaborate these results. Finally, regression analyses examine the associations of perceived physician guidance and trust with clinical leadership, and mediation analysis examines the indirect association between perceived physician trust and clinical leadership through perceived physician guidance. Qualitative findings provide additional context for these associations. Qualitative quotations are labeled using the format ID###‐Stage‐Setting to indicate participant identification number, career stage (T1/T2; P0|1 to P4|5), and work setting (PC = primary care; TC = tertiary care). For example, NP134‐P4|5‐TC refers to participant 134, an NP with four to five years of practice experience who worked in tertiary care.

### 3.1. Descriptive Results

A total of 300 early‐career NPs participated in the study, of whom 222 fully completed questionnaires and were included in the analysis. Participants represented a broad age range, with 22.5% aged over 50 years. Nearly half were in training, and 23.4% had 4‐5 years of NP practice experience. Prior to NP training, most participants reported substantial nursing experience, with 36.9% having worked as registered nurses for more than 17 years (Table 1).

**TABLE 1:** Participant characteristics (*N* = 222).

Variables	*n* (%)
Age	
≤ 30	32 (14.4%)
31–35	40 (18%)
36–40	38 (17.1%)
41–45	28 (12.6%)
46–50	34 (15.3%)
> 50	50 (22.5%)
Stage of career	
First year of training	55 (24.8%)
Second year of training	45 (20.3%)
0–1 year in practice	17 (7.7%)
1–2 years in practice	17 (7.7%)
2–3 years in practice	24 (10.8%)
3–4 years in practice	12 (5.4%)
4–5 years in practice	52 (23.4%)
Previous nursing experience (years)	
2–5 Years	39 (17.6%)
6–9 Years	41 (18.5%)
10–13 Years	35 (15.8%)
14–17 Years	25 (11.3%)
> 17 Years	82 (36.9%)
Specialization during training	
General healthcare	186 (83.8%)
Mental healthcare	36 (16.2%)
Work setting	
Primary care	94 (42.3%)
Tertiary care	128 (57.7%)
Team size	
≤ 6	50 (22.5%)
7–15	96 (43.2%)
> 15	76 (34.2%)
Perceived team affiliation	
Medical team	149 (67.1%)
Medical & Nursing team	35 (15.8%)
Nursing team	38 (17.1%)
Perceived clarity of NP role among team members	
Low clarity	36 (16.2%)
Moderate clarity	26 (11.7%)
High clarity	160 (72.1%)
Perceived collaboration among team members^a^	
Physicians	205 (92%)
Nurses	173 (78%)
Nurse practitioners	131 (59%)
Doctors’ assistants	75 (34%)
Administrative staff	68 (31%)
Practice managers	48 (22%)
Physician assistants	29 (13%)

^a^Physicians include residents too.

Most participants specialized in general healthcare during their training (83.8%). Participants worked in primary (42.3%) or tertiary care settings (57.7%). Team size varied, with 43.2% working in teams comprising 7–15 members. Most respondents (67.1%) identified primarily with being a member of a medical team, while others associated with nursing team membership (17.1%) or both (15.8%). In addition, 72.1% perceived that their NP role was well understood by other members of the healthcare team. NPs reported collaborating with a range of healthcare professionals, most commonly with physicians (92%), followed by nurses (78%) and other NPs (59%).

### 3.2. Clinical Leadership Among NPs in Their Early Career

As shown in Table 2, participants in the first training year had the lowest overall clinical leadership score (3.94 ± 0.34), whereas the highest score was reported by those with 4–5 years of experience (4.23 ± 0.35). ANOVA results indicated significant group differences, with NPs in the 2–3 and 4–5 years postgraduation groups reporting higher overall leadership scores than those in their first training year (Table 3).

**TABLE 2:** Clinical leadership scores by stages of early career.

Early career stage	Overall leadership	Visionary and inspirational role modeling	Empowering and supporting others	Reflective and development‐oriented practice
First year of training	3.94 ± 0.34	3.72 ± 0.44	4.16 ± 0.43	3.94 ± 0.54
Second year of training	4.11 ± 0.34	4.03 ± 0.48	4.15 ± 0.40	4.21 ± 0.43
0–1 year in practice	4.22 ± 0.38	4.09 ± 0.41	4.26 ± 0.50	4.41 ± 0.59
1–2 years in practice	4.08 ± 0.34	4.01 ± 0.53	4.14 ± 0.39	4.12 ± 0.52
2–3 years in practice	4.21 ± 0.39	4.12 ± 0.51	4.36 ± 0.39	4.08 ± 0.52
3–4 years in practice	4.20 ± 0.45	4.13 ± 0.66	4.23 ± 0.51	4.29 ± 0.45
4–5 years in practice	4.23 ± 0.35	4.22 ± 0.51	4.25 ± 0.38	4.22 ± 0.44

**TABLE 3:** Clinical leadership scores by demographic and professional characteristics.

Variable	Mean ± SD	Test	*p*	Significant pairs
Age				
≤ 30	3.95 ± 0.30	ANOVA	**< 0.001**	≤ 30–> 50; 36–40–> 50; 46–50–≤ 30
31–35	4.05 ± 0.37			
36–40	4.02 ± 0.33			
41–45	4.10 ± 0.34			
46–50	4.25 ± 0.34			
> 50	4.27 ± 0.41			
Stage of career				
First year of training	3.94 ± 0.34	ANOVA	**0.001**	2–3 years in practice—First year of training; 4–5 years in practice—First year of training
Second year of training	4.11 ± 0.34			
0–1 year in practice	4.22 ± 0.38			
1–2 years in practice	4.08 ± 0.34			
2–3 years in practice	4.21 ± 0.39			
3–4 years in practice	4.20 ± 0.45			
4–5 years in practice	4.23 ± 0.35			
Previous nursing experience (years)				
2–5 Years	4.04 ± 0.38	ANOVA	**0.022**	
6–9 Years	4.08 ± 0.31			
10–13 Years	4.10 ± 0.39			
14–17 Years	4.00 ± 0.32			
> 17 Years	4.22 ± 0.39			
Specialization during training				
General healthcare	4.08 ± 0.35	*t*‐test	**< 0.001**	
Mental healthcare	4.33 ± 0.38			
Work setting				
Primary care	4.08 ± 0.39	*t*‐test	0.226	
Secondary care	4.14 ± 0.35			
Team size				
≤ 6	4.10 ± 0.33	ANOVA	0.785	
7–15	4.11 ± 0.36			
> 15	4.14 ± 0.40			
Perceived team affiliation				
Medical team	4.11 ± 0.38	ANOVA	0.891	
Medical & Nursing team	4.12 ± 0.37			
Nursing team	4.14 ± 0.35			
Perceived clarity of NP role among team members				
Low clarity	4.11 ± 0.36	ANOVA	0.270	
Moderate clarity	4.02 ± 0.36			
High clarity	4.14 ± 0.37			

*Note:* Values are presented as mean ± standard deviation (SD). Differences between groups were examined using one‐way ANOVA or independent‐samples *t*‐tests. Significant results are indicated in bold (*p* < 0.05).

For *the visionary and inspirational role-modeling* dimension, first‐year student NPs reported the lowest mean score (3.72 ± 0.44), while higher mean scores were observed among NPs with one to two years (4.01 ± 0.53) and four to five years of practice experience (4.22 ± 0.51). Student NPs described, “*I cannot yet position myself as a leading professional*” (NP282‐T1‐PC). In contrast, NPs in later career‐stage groups reported “*taking initiative where there is no clear vision*” (NP024‐P0‐1‐PC) and “*directing co-practitioners and assuming accountability for care*” (NP134‐P4|5‐TC). The dimension *empowering and supporting others* showed consistently high mean scores across groups (4.14–4.36), with the highest scores observed among NPs with 2–3 years of practice experience (4.36 ± 0.39). Participants who were still in training described early efforts to facilitate team collaboration, such as “*involving the team and motivating colleagues*” (ID219‐T1‐TC). NPs in the practice‐stage groups described “*being accessible and coaching*” (ID050‐P0|1‐TC), “*empowering the palliative care nursing team*” (ID127‐P2‐3‐PC), and “*motivating others and letting others shine*” (ID135‐P4|5‐PC). Scores for *reflective and development-oriented practice* also differed across career‐stage groups, with the lowest mean score reported by first‐year student NPs (3.94 ± 0.54) and the highest by NPs in their first year of practice (4.41 ± 0.59). Reflective practice described by student NPs was more basic, for example, “*critically reviewing working methods*” (ID088‐T1‐TC). NPs with more practice experience described more routine, reflective, and development‐oriented practice and tied it to quality improvement: “*demonstrating reflective ability and developing protocols*” (ID165‐P3|4‐PC), “*engaging in evidence-based practice with a reflective attitude*” (ID220‐P4|5‐PC).

As shown in Table 3, clinical leadership differed significantly by age, prior nursing experience, and specialization in mental healthcare. No significant differences were observed by work setting, team size, team affiliation, or perceived role clarity within teams. Qualitative findings showed that older and more experienced NPs described greater confidence and influence within team collaborations, noting that “*colleagues rely on me and ask me to join improvement projects*” (ID073‐P4|5‐PC). In contrast, less experienced NPs emphasized ongoing development of clinical competence, describing how they were “*taking responsibility and showing initiative*” (ID125‐P0|1‐PC). Among NPs specializing in mental healthcare, participants tended to describe more autonomous roles, such as “*establishing and developing this* [NP] *discipline with the psychiatrist*” (ID137‐T1‐TC).

### 3.3. The Association of Physicians’ Guidance and Trust on NPs Clinical Leadership

In the multiple linear regression model (Table 4), perceived physician guidance was significantly and positively associated with leadership (*β* = 0.139, SE = 0.037, *p* < 0.001), whereas perceived physician trust was not significantly associated with clinical leadership (*β* = −0.037, SE = 0.036, *p* = 0.315).

**TABLE 4:** Multiple linear regression examining the influence of perceived physicians guidance and trust on clinical leadership among early‐career NPs.

Variable	Estimate	SE	*p*
(Intercept)	3.639^∗∗∗^	0.216	**< 0.001**
Perceived guidance from physicians	0.139^∗∗∗^	0.037	**< 0.001**
Perceived trust from physicians	−0.037	0.036	0.315
Age			
≤ 30	[reference]		
31–35	−0.225	0.130	0.085
36–40	−0.203	0.108	0.060
41–45	−0.194^∗^	0.098	0.049
46–50	−0.159	0.088	0.073
> 50	0.034	0.084	0.685
Stage of career			
First year of training	[reference]		
Second year of training	0.174^∗^	0.072	**0.016**
0–1 year in practice	0.246^∗^	0.101	**0.016**
1–2 years in practice	0.160	0.105	0.130
2–3 years in practice	0.224^∗^	0.092	**0.016**
3–4 years in practice	0.128	0.125	0.307
4–5 years in practice	0.182^∗^	0.083	**0.030**
Previous nursing experience (years)			
2–5 Years	[reference]		
6–9 Years	0.061	0.092	0.505
10–13 Years	−0.126	0.090	0.162
14–17 Years	0.047	0.106	0.657
> 17 Years	0.043	0.100	0.670
Specialization during training			
General healthcare	[reference]		
Mental healthcare	0.167^∗^	0.070	**0.018**
Work setting			
Primary care	[reference]		
Secondary care	0.038	0.056	0.504
Team size			
≤ 6	[reference]		
7–15	−0.071	0.067	0.286
> 15	−0.057	0.072	0.430
Perceived team affiliation			
Medical team	[reference]		
Medical & Nursing team	0.032	0.072	0.660
Nursing team	0.005	0.087	0.951
Perceived clarity of NP role among team members			
Low clarity	[reference]		
Moderate clarity	−0.003	0.092	0.972
High clarity	0.003	0.071	0.967

*Note:* Significance levels: ^∗∗∗^ *p* < 0.001; ^∗^ *p* < 0.01. The bold values indicate statistically significant results.

Mediation analysis (Table 5) indicated a significant indirect association between perceived physician trust and clinical leadership through perceived physician guidance (ACME = 0.039, 95% CI [0.012, 0.078], *p* = 0.002), while both the direct and total effects were nonsignificant (ADE = −0.036, *p* = 0.342; total effect = 0.003, *p* = 0.932), indicating full mediation. Regression paths supported this model: perceived physician trust was positively associated with perceived guidance, which was, in turn, positively associated with clinical leadership (Table S6 in Supporting Information).

**TABLE 5:** Mediation analysis examining the indirect influence of perceived physician trust on NPs’ clinical leadership through perceived physician guidance.

Effect	Estimate	95% CI lower	95% CI upper	*p*
ACME (indirect)	0.039	0.012	0.078	0.002
ADE (direct)	−0.036	−0.111	0.038	0.342
Total effect	0.003	−0.077	0.075	0.932
Proportion mediated	13.067			

Abbreviations: ACME, average causal mediation effect; ADE, average direct effect.

The following section presents a qualitative analysis of the open‐ended responses concerning how perceived physician guidance and trust might support or constrain their clinical leadership.

Through thematic analysis, we identified three ways of guidance from physicians that NPs perceived as supporting their clinical leadership: emotional and practical support, constructive feedback, and opportunities to enact leadership. First, emotional and practical support was perceived as supporting professional growth and psychological safety for NPs’ clinical leadership development. One NP described feeling fully supported: *“Physicians stood beside me and supported me with everything…* both *during and outside working hours”* (ID52‐P3|4‐PC). Second, constructive feedback was described as supporting reflective learning. NPs valued constructive GP feedback, such as *“GP think along and let me look at things from another perspective”* (ID24‐P0|1‐PC). NPs appreciated kind, supportive delivery of feedback: *“They phrase it gently, understand if I cannot do something, and encourage me kindly”* (ID236‐T2‐TC). Third, opportunities to enact leadership through trust, delegation, and formal roles were perceived as supporting NPs’ clinical leadership. Some were trusted with *“making medical decisions”* (ID50‐P0|1‐TC), while others described more formal leadership roles: *“I was encouraged to set up a joint daily board to give vision and direction to the care chain”* (ID143‐P4|5‐TC).

Participants described different functions of physicians’ guidance across career‐stage groups. During training, guidance was described as a form of scaffolding, with physicians providing support, observation, and feedback to facilitate learning: *“debrief before and after conversations and* [they] *observe me during consultations, which helps me grow”* (ID075‐T1‐TC). Among NPs with up to three years of practice experience, guidance was most often described in terms of professional legitimation, with perceived physician trust and recognition creating opportunities to assume leadership roles. For example, NPs noted that *“doctors ask me for advice in my area of expertise, which makes me dare to take on my leadership role”* (ID051‐P2‐3‐TC), and that they were treated as *“an equal colleague whose input is valued and implemented”* (ID060‐P3‐4‐TC). In comparison, NPs with four to five years of practice experience more often described guidance in terms of strategic partnership, including involvement in organizational‐ and system‐level initiatives: *“doctors regularly refer questions to me and involve me in shaping our service”* (ID156‐P4‐5‐TC).

In contrast, the absence of physicians’ guidance was described as a barrier to NPs’ leadership development. Participants reported hierarchical dynamics, role uncertainty, and limited feedback, which was perceived as limiting their opportunities to lead. As one NP noted, *“Some doctors think they are the ‘only specialists’ and that NPs must always work under supervision”* (ID018‐T2‐PC). Another explained that *“doctors are hesitant to share responsibilities or do not fully understand or acknowledge the NP role which limits my ability to take a leading and coordinating role”* (ID246‐T1‐PC). Time pressures and heavy workloads were also described as restricting access to supervision: *“Because I am the first and only one, and the doctors are very busy, it is sometimes difficult to get support from them”* (ID193‐P2|3‐TC).

The qualitative findings suggested that participants described trust as an important condition for enacting clinical leadership, although the quantitative data did not show a significant association. NPs described trust as contributing to psychological safety and autonomy. They noted that trust “*gives me the confidence to raise issues for improvement*” (ID211‐T2‐PC) or “*makes me dare to take the lead*” (ID033‐P4|5‐TC). Importantly, trust translated into leadership opportunities when accompanied by physicians’ support: “*The doctors give us full trust to take and perform our role as medical coordinating practitioners*” (ID203‐P1|2‐TC). However, participants described uneven trust across physicians as potentially constraining clinical leadership: “*Some doctors have a lot of trust, which allows me to take leadership. I conduct the consultation and coordinate the care. Others have less trust, which hinders my leadership roles*” (ID151‐P0|1‐TC).

### 3.4. Synthesis of Findings

Our mixed‐methods findings suggest that clinical leadership among early‐career NPs is associated not only with career stage and prior experience but also with relational conditions such as perceived physician guidance and trust. Quantitatively, leadership scores were higher among older NPs, those with extensive prior nursing experience, and those with more years of NP practice. The qualitative findings elaborated on these patterns by showing how NPs perceived physician guidance and trust as shaping their clinical leadership. NPs specializing in mental healthcare reported particularly high clinical leadership scores—a finding echoed qualitatively in descriptions of their experience of having more autonomy on the team.

The quantitative analyses identified perceived physician guidance as being directly associated with clinical leadership. Additional insights from the qualitative findings showed that NPs perceived physician guidance provided the scaffolding, legitimization, and eventually partnership that allowed NPs to enact clinical leadership roles in practice. Perceived physician trust, however, was not directly associated with clinical leadership in the quantitative model. However, the exploratory mediation analysis indicated an indirect association through guidance, while qualitative analysis suggested that trust contributed to psychological safety, professional legitimacy, and opportunities for initiative, thereby creating conditions in which guidance could support clinical leadership.

## 4. Discussion

### 4.1. Summary of Findings

This cross‐sectional mixed‐methods study examined clinical leadership among early‐career NPs in relation to perceived physician guidance and trust. Overall, NPs reported moderate to high levels of clinical leadership, with more experienced NPs reporting higher clinical leadership scores. Quantitative findings further demonstrated that perceived physician guidance was significantly associated with clinical leadership, while perceived physician trust was not directly associated with clinical leadership. Exploratory mediation analysis indicated an indirect association between trust and clinical leadership through guidance. Qualitative findings supported these patterns by showing that NPs perceived physicians’ emotional and practical support, constructive feedback, and provision of leadership opportunities helped NPs build confidence, reflective capacity, and professional legitimacy. They also described trust from physicians as contributing to psychological safety and NPs’ professional autonomy, which enabled their guidance to support NPs’ leadership development.

### 4.2. Comparison With Previous Findings

Our findings suggest that NPs in early practice reported higher levels of clinical leadership than those in training. Consistent with prior research on NP role transition, early‐stage NPs reported limited clinical competence, uncertainty regarding role responsibilities, and a lack of professional legitimacy [6, 8, 55], all of which may constrain their ability to enact clinical leadership. Across career‐stage groups, greater clinical competence was accompanied by greater perceived legitimacy and authority to assume leadership roles within healthcare teams. These findings suggest that NP clinical leadership is not solely an individual competency but a situated and relational practice that develops through interactions with other health professionals. This perspective is consistent with situated learning theory, which conceptualizes workplace learning as occurring through everyday practice and interprofessional engagement [16, 17].

Our findings indicate that NP clinical leadership was positively associated with perceived physician guidance, while perceived physician trust showed an indirect association with clinical leadership through guidance. Through the lens of sociocultural learning theories [16, 17], such guidance plays a critical role in informal and implicit workplace learning [24–26]. In our study, NPs described physicians’ practical and emotional support and constructive feedback as supporting their clinical leadership. Guidance was described differently across career‐stage groups: NPs in training emphasized close scaffolding, whereas those in practice more often described professional legitimation. Previous research suggests that physician trust may further support these workplace learning conditions by recognizing NPs as competent members of the practice community, thereby facilitating greater autonomy and sustained involvement in leadership activities [21, 23, 33, 35, 56–58]. Importantly, our findings suggest that trust was not directly associated with clinical leadership but may have created relational conditions in which guidance and clinical leadership could be enacted. However, these findings are situated within the Dutch NP training context, where NPs have traditionally been educated under physician supervision, although an increasing number are now also trained by experienced NPs. This historical positioning may help explain why physician guidance was often perceived as supervisory and why many NPs experienced a partial belonging to the physician community, reinforcing physicians’ role in shaping leadership opportunities during training and early practice. These findings may differ across healthcare systems and healthcare settings. For example, in parts of the United States, NP education is predominantly NP‐led and professional autonomy is higher in primary care [59], whereas in the United Kingdom, NP‐led education coexists with strong IPC, which may sustain the importance of physician guidance and trust in clinical leadership development [3].

### 4.3. Practical Implications

This study provides practical implications for supporting clinical leadership among early‐career NPs. Given that clinical leadership mainly develops through workplace participation, NP education programs can incorporate explicit clinical leadership development into their curricula and prepare NPs for progressively greater responsibility [24–26]. This preparation should be reinforced in clinical workplaces through structured supervision during training and early practice, enabling NPs to observe physician leadership, receive feedback, and safely practice leadership tasks. To strengthen these workplace learning conditions, preparation of physicians for supervisory roles should be incorporated into continuing professional development, with an emphasis on developmentally appropriate guidance and constructive feedback that may support NPs’ clinical leadership. In addition, trust‐based relationships also appear to be an important relational condition for clinical leadership. Physicians may foster trust in NPs by involving NPs in clinical reasoning and recognizing their contributions within teams [21, 23, 33, 35, 56–58]. NPs, in turn, may contribute to developing physicians’ trust by demonstrating clinical preparedness, communicating openly about uncertainties, and seeking feedback proactively.

### 4.4. Limitations

Several limitations should be acknowledged. First, the cross‐sectional design limits causal interpretation. Longitudinal studies are therefore needed to examine changes in NPs’ clinical leadership over time and the relationships among clinical leadership, perceived physician guidance, and perceived physician trust; qualitative studies could also provide valuable insights into NPs’ clinical leadership behaviors and the role of physicians in the development of NPs’ clinical leadership. Second, the reliance on self‐reported data and voluntary participation may have introduced recall, social desirability, and self‐selection biases, potentially affecting the representativeness of the sample and the robustness of the findings [60]. Third, CFA was not conducted using an independent validation sample due to practical constraints and the relatively small population of early‐career NPs in the Netherlands. In addition, the absence of a practicing NP in our research team might have limited the profession‐specific perspectives considered during data analysis and interpretation of the findings. Future studies should therefore validate the adapted questionnaire in larger and more diverse samples and involve NPs as research collaborators throughout the research process. Finally, differences in program structures, supervision practices, and organizational cultures across countries may limit the transferability of the findings.

## 5. Conclusion

Findings from this cross‐sectional mixed‐methods study in the Dutch context indicate that NP clinical leadership varies across stages of training and early practice and is shaped by relational processes with physicians in clinical settings. Perceived physician guidance was important to clinical leadership development, while trust enabled this guidance by fostering psychological safety and NPs’ clinical autonomy. These findings highlight physicians’ role in providing developmentally appropriate guidance, constructive feedback, and graduated leadership opportunities, as well as fostering trust‐based relationships that enable NPs’ autonomy and clinical leadership during early practice.

## Author Contributions

Guoyang Zhang: conceptualization; methodology; validation; formal analysis; data curation; writing–original draft; writing–review and editing.

Hester W. H. Smeets: conceptualization; validation; investigation; resources; writing–review and editing.

Carolin Sehlbach: conceptualization; methodology; validation; supervision; writing–review and editing.

Frank W. J. M. Smeenk: conceptualization; methodology; validation; investigation; resources; supervision; writing–review and editing.

Renée E. Stalmeijer: conceptualization; methodology; validation; investigation; resources; supervision; writing–review and editing.

## Funding

This work was supported by the China Scholarship Council under Grant number NO.202208310033.

## Disclosure

This funder had no role in the design and development of the study or the decision to publish.

## Ethics Statement

This research was approved by the Ethical Review Committee of the Faculty of Health, Medicine and Life Sciences at Maastricht University (reference number FHML‐REC/2024/078). All participants gave written consent.

## Conflicts of Interest

The authors declare no conflicts of interest.

## Supporting Information

Additional supporting information can be found online in the Supporting Information section.

## Supporting information


**Supporting Information 1** The Supporting Information includes the English‐language questionnaire and six supporting tables presenting the questionnaire’s psychometric validation (Tables S1–S5) and mediation analysis results (Table S6).

**Supporting Information 2** Reporting Guideline Checklist.

## Data Availability

Anonymized data supporting the findings of this study are available from the corresponding author upon reasonable request.
