# Supplementary material for: The Influence of Perceived Physician Guidance and Trust on Early‐Career Nurse Practitioners’ Clinical Leadership: A Cross‐Sectional Mixed‐Methods Study
Source: J Nurs Manag. 2026 Nov 3;2026:1900444. doi: 10.1155/jonm/1900444 (PMC13729086; doi:10.1155/jonm/1900444)
Supplement: Supplementary file 1 [file JONM-2026-1900444-s001.docx]

## Supplementary Material

The Supplementary Materials include the English-language questionnaire and six supplementary tables presenting the questionnaire’s psychometric validation (Tables S1–S5) and mediation analysis results (Table S6).

### Questionnaire (English version)

Dear participant,

Thank you for participating in this survey!

With this survey, we want to investigate how nurse practitioners (in training) develop their leadership competencies during their training and in the first five years after completing their training. We are also curious about the role the physicians in your care team play in this competency development.

**Who can participate?**

Nurses in training to become specialists and nurse practitioners who have completed their training and have up to five years of work experience.

**Why is this important?**

Your input helps us improve leadership training and interprofessional collaboration in practice.

**Your privacy**

Participation is voluntary, and your responses will be kept confidential.

The survey will take approximately 10–15 minutes. If you have any questions, please contact us by email. By continuing, you consent to participate in this study. Thank you!

Guoyang Zhang, Hester Smeets, Frank Smeenk, Carolin Sehlbach, Renée Stalmeijer

Maastricht University, Zuyd University of Applied Sciences

1. What is your age?

- < 26 years
- 26–30 years
- 31–35 years
- 36–40 years
- 41–45 years
- 46–50 years
- 51–55 years
- 56–60 years
- > 60 years

2. I am:

- Nurse Practitioner (NP) student → proceed to 2a, 4a, 5a, 7a, 11a
- Nurse Practitioner → proceed to 2b, 4b, 5b, 7b, 7b2, 7b3, 11b

2a. In which year of the program are you currently enrolled?

- 1st year
- 2nd year

2b. How many years of experience do you have as a Nurse Practitioner?

- 0–1 year
- 1–2 years
- 2–3 years
- 3–4 years
- 4–5 years

3. How many years of nursing experience did you have before starting the NP training program?

- 2–5 years
- 6–9 years
- 10–13 years
- 14–17 years
- > 17 years

4. 4a. At which university of applied sciences are you currently following the Master of Advanced Nursing Practice?

4b. At which university of applied sciences did you complete the Master of Advanced Nursing Practice?

- Fontys University of Applied Sciences
- Hanze University of Applied Sciences Groningen
- HAN University of Applied Sciences (Arnhem Nijmegen)
- Leiden University of Applied Sciences
- Rotterdam University of Applied Sciences
- Saxion University of Applied Sciences
- Utrecht University of Applied Sciences
- Inholland University of Applied Sciences (Amsterdam)
- Zuyd University of Applied Sciences

5. 5a. Which specialization are you following in your NP training?

5b. Which specialization did you follow during your NP training?

- General Healthcare
- Mental Healthcare

6. In which healthcare setting do you mainly work?

- Primary care / General practice
- Nursing home / Residential care / Home care (VVT)
- Mental health care – primary care
- Mental health care – secondary care
- Disability care
- Occupational / Public health
- Emergency care
- Hospital / Secondary care
- Other, namely: _______

7. 7a. Who are members of your training team? (multiple answers possible)

- Master of Advanced Nursing Practice Coach
- Clinical Supervisor – Physician
- Clinical Supervisor – Nurse Practitioner
- Clinical Supervisor – Other, namely: _______
- Practical Trainer – Physician
- Practical Trainer – Physician Assistant (PA)
- Practical Trainer – Nurse Practitioner
- Practical Trainer – Other, namely: _______
- Manager – Physician
- Manager – PA
- Manager – NP
- Manager – Other, namely: _______

7b1. Who is your manager/supervisor—the person to whom you report?

- Physician
- Nurse Practitioner
- Manager
- Other, namely: _______

7b2. Does your healthcare organization require supervision?

- Yes → proceed to 7b3
- No

7b3. Who is your supervisor?

- Physician
- Nurse Practitioner
- Manager
- Other, namely: _______

8. What is the size of the team in which you primarily work?

(Note: “Team” refers to the group of people with whom you directly and regularly collaborate in providing patient care.)

- Fewer than 3 people
- 3–6 people
- 7–10 people
- 11–14 people
- 15–18 people
- More than 18 people, namely: _______

9. With which professional groups do you primarily collaborate in patient care?

- Administrative staff
- Medical assistant
- General practitioner
- (Practice/Department) Manager
- Medical specialist
- Practice nurse
- Psychiatric nurse
- Registered nurse
- Nurse practitioner
- Physician assistant
- Other, namely: _______

10. I am assigned within the:

- Nursing team
- Medical team

11. 11a. Within the team, there is sufficient clarity about my scope of practice as an NP student.

- Strongly disagree
- Disagree
- Neutral
- Agree
- Strongly agree

11b. Within the team, there is sufficient clarity about my scope of practice as a Nurse Practitioner.

- Strongly disagree
- Disagree
- Neutral
- Agree
- Strongly agree

12. In the following statements, we aim to gain insight into how you perceive your leadership within your team. Nursing leadership can be defined as “the systematic demonstration of leadership behaviors that influence others—patients, colleagues, and teams—to achieve shared goals in patient care.” The following items are adapted from a previously validated questionnaire and modified for the purposes of this study. Please rate yourself on a scale from 1 (Almost never) to 5 (Almost always).
1) I try to set a personal example by being a role model in the team.
2) I actively contribute to improving patient care.
3) I follow through on promises.
4) I ask team members for feedback on how my actions impact their performance.
5) I am committed to providing patient-centered care.
6) I have a clear vision of how to address the challenges of future healthcare.
7) I try to engage others to share their visions of future healthcare.
8) I motivate team members to align their work with patient-centered care.
9) I seek opportunities to learn and grow professionally.
10) I encourage team members to try new approaches to enhance patient care.
11) I apply evidence-based practice principles in patient care.
12) I reflect on ways in which I can improve my work.
13) I actively listen to team members’ ideas and concerns.
14) I involve team members in making decisions.
15) I try to foster a collaborative environment in which we trust each other.
16) I help team members grow their competencies.
17) I celebrate team members’ achievements.
18) I show that I trust my team members to do their jobs well.
19) I recognize and appreciate team members’ contributions.
20) I offer constructive feedback to team members.
21) Open-ended question: What demonstrates your leadership in day-to-day healthcare practice. Please list 3 examples.
13. Trust

(1) To what extent do you experience that the physicians in your team trust you in providing patient care?

- Not at all
- A little
- Somewhat
- Much
- Completely

(2) In what way does the degree of trust that physicians place in you influence how you enact your leadership role within the team? (open-ended question)

14. Physicians’ Support

To what extent do you agree with the following statements regarding the support you experience from physicians in your leadership development? (1 = Strongly disagree to 5 = Strongly agree). The physicians (all physicians, excluding medical students, interns, or senior residents) in my team...

1) Emphasize that I can approach them when I have questions.
2) Provide me with (practical) support when I need it.
3) Provide me with emotional support when I need it.
4) Encourage me to take initiative in patient care that aligns with my expertise.
5) Give me constructive feedback.
6) Provide explanations on how I can improve my performance.
15. Open-ended Questions on Support
1) In what ways do you experience support from physicians when carrying out your leadership role within the team? Could you provide an example?
2) To what extent do you experience a lack of support for this? Could you provide an example?

### Table S1 Kaiser-Meyer-Olkin factor adequacy-20 items

| **Items** | **MSA** |
| --- | --- |
| I try to set a personal example by being a role model in the team. | 0.88 |
| I actively contribute to improving patient care. | 0.54 |
| I follow through on promises. | 0.49 |
| I ask team members for feedback on how my actions impact their performance. | 0.78 |
| I am committed to providing patient-centered care. | 0.43 |
| I have a clear vision of how to address the challenges of future healthcare. | 0.51 |
| I try to engage others to share their visions of future healthcare. | 0.44 |
| I motivate team members to align their work with patient-centered care. | 0.88 |
| I seek opportunities to learn and grow professionally. | 0.78 |
| I encourage team members to try new approaches to enhance patient care. | 0.89 |
| I apply evidence-based practice principles in patient care. | 0.84 |
| I reflect on ways in which I can improve my work. | 0.7 |
| I actively listen to team members’ ideas and concerns. | 0.61 |
| I involve team members in making decisions. | 0.78 |
| I try to foster a collaborative environment in which we trust each other. | 0.61 |
| I help team members grow their competencies. | 0.64 |
| I celebrate team members’ achievements. | 0.66 |
| I show that I trust my team members to do their jobs well. | 0.44 |
| I recognize and appreciate team members’ contributions. | 0.49 |
| I offer constructive feedback to team members. | 0.95 |
| **Overall KMO** | 0.63 |

**Note**. Item-level measures of sampling adequacy (MSA) were calculated using the Kaiser–Meyer–Olkin (KMO) test to evaluate the suitability of the data for factor analysis. The overall KMO value of 0.63 indicated a mediocre level of sampling adequacy, suggesting that some items might not be optimally suited for factor extraction. Items with MSA values below 0.50, or with substantial cross-loadings or low factor loadings, were excluded from further analysis (items highlighted in red).

### Table S2 Kaiser-Meyer-Olkin factor adequacy-12 items

| Items | MSA for each item |
| --- | --- |
| I try to set a personal example by being a role model in the team. | 0.86 |
| I actively contribute to improving patient care. | 0.82 |
| I have a clear vision of how to address the challenges of future healthcare. | 0.81 |
| I motivate team members to align their work with patient-centered care. | 0.88 |
| I encourage team members to try new approaches to enhance patient care. | 0.82 |
| I apply evidence-based practice principles in patient care. | 0.75 |
| I reflect on ways in which I can improve my work. | 0.73 |
| I actively listen to team members’ ideas and concerns. | 0.74 |
| I involve team members in making decisions. | 0.86 |
| I try to foster a collaborative environment in which we trust each other. | 0.79 |
| I celebrate team members’ achievements. | 0.89 |
| I show that I trust my team members to do their jobs well. | 0.88 |

**Note.** The Kaiser–Meyer–Olkin (KMO) measure of sampling adequacy for the 12-item scale was 0.82, indicating a good level of suitability for exploratory factor analysis.

### Table S3 Factor Loadings for Final 12 Items

| Items | Visionary and Inspirational Role Modeling | Empowering and Supporting Others | Reflective and Development-Oriented Practice |
| --- | --- | --- | --- |
| I try to set a personal example by being a role model in the team. | 0.59 |  |  |
| I actively contribute to improving patient care. | 0.83 |  |  |
| I have a clear vision of how to address the challenges of future healthcare. | 0.78 |  |  |
| I motivate team members to align their work with patient-centered care. | 0.52 |  |  |
| I encourage team members to try new approaches to enhance patient care. | 0.73 |  |  |
| I apply evidence-based practice principles in patient care. |  |  | 0.46 |
| I reflect on ways in which I can improve my work. |  |  | 0.97 |
| I actively listen to team members’ ideas and concerns. |  | 0.75 |  |
| I involve team members in making decisions. |  | 0.44 |  |
| I try to foster a collaborative environment in which we trust each other. |  | 0.78 |  |
| I celebrate team members’ achievements. |  | 0.65 |  |
| I show that I trust my team members to do their jobs well. |  | 0.64 |  |

**Note.** An exploratory factor analysis (EFA) using maximum likelihood extraction with oblimin rotation yielded a clear three-factor solution. Factor 1, labelled *Visionary and Inspirational Role Modeling*, comprised five items (factor loadings = 0.52–0.83) that reflected role modeling, vision, and motivating others toward patient-centred care. Factor 2, *Empowering and Supporting Others*, included six items (loadings = 0.44–0.78) representing collaboration, trust, and the active support of team members’ development. Factor 3, *Reflective and Development-Oriented Practice*, consisted of two items (loadings = 0.46–0.97) capturing reflective practice and professional growth. Together, the three factors explained approximately 56% of the total variance, indicating an interpretable and theoretically coherent factor structure among early-career NPs.

### Table S4 Reliability Scores for the Questionnaire

| Factor | Cronbach_alpha |
| --- | --- |
| Visionary and Inspirational Role Modeling | 0.79 |
| Empowering and Supporting Others | 0.73 |
| Reflective and Development-Oriented Practice | 0.58 |

**Note**. Cronbach’s α coefficients were calculated to assess the internal consistency reliability of each subscale among early-career NPs. The α values ranged from 0.58 to 0.79, indicating acceptable to good internal consistency. The two-item subscale (*Reflective and Development-Oriented Practice*) demonstrated a lower α value, which is considered acceptable given its brevity.

### Table S5 Split-half / Resampling-based Factorial Stability (Tucker’s φ)

| Factor | Mean_Tucker_phi | Q25 | Median | Q75 |
| --- | --- | --- | --- | --- |
| Visionary and Inspirational Role Modeling | 0.919 | 0.907 | 0.930 | 0.95 |
| Empowering and Supporting Others | 0.915 | 0.898 | 0.920 | 0.95 |
| Reflective and Development-Oriented Practice | 0.679 | 0.568 | 0.675 | 0.83 |

**Note.** Tucker’s coefficient of congruence (φ) was calculated using split-half resampling to assess factorial stability across random subsamples. Mean φ values above 0.85 indicate high stability, values between 0.68–0.85 indicate moderate stability.

### Table S6 Regression paths for mediation model

| **Path** | **B (Unstandardized)** | **β (Standardized)** |
| --- | --- | --- |
| Trust → Guidance (a) | 0.276 | 0.308 |
| Guidance → Leadership (b) | 0.140 | 0.260 |
| Trust → Leadership (c′) | -0.031 | -0.064 |

**Note.** Path *a* represents the effect of physicians’ trust on guidance, path *b* represents the effect of guidance on leadership, and path *c′* represents the direct effect of trust on leadership after accounting for guidance. The results showed that trust significantly predicted guidance (*B* = 0.276, β = 0.308), and guidance significantly predicted leadership (*B* = 0.140, β = 0.260). However, the direct effect of trust on leadership was not significant (*B* = −0.031, β = −0.064). These findings indicate that physicians’ trust influenced leadership indirectly through its positive association with guidance, supporting the mediation effect identified in the model.
